# Efficient Topical Treatment of Canine Nodular Sebaceous Hyperplasia with a Nitric Acid and Zinc Complex Solution

**DOI:** 10.3390/ani14040570

**Published:** 2024-02-08

**Authors:** Lina Gustafsson, Alison Wilson, Kerstin Bergvall

**Affiliations:** 1 Evidensia Södra Djursjukhuset, Månskärsvägen 13, 141 75 Huddinge, Sweden; alison.wilson@evidensia.se (A.W.); or kerstin.bergvall@evidensia.se (K.B.); 2 Swedish University of Agricultural Sciences, Faculty of Veterinary Medicine and Animal Science, P.O. Box 7084, 750 07 Uppsala, Sweden

**Keywords:** epithelioma, nodular sebaceous gland hyperplasia, sebaceous gland adenoma, sebaceous tumors, topical treatment

## Abstract

**Simple Summary:**

Nodular sebaceous gland hyperplasia refers to wartlike, hairless nodules that are common in middle-aged to old dogs. If it disturbs the dog, it is usually removed by surgical excision. As anesthesia is associated with risk, which is increased in older patients, an alternative to surgery is desirable. A solution containing nitric acid, zinc, copper, and organic acids (Verrutop^®^, ISDIN, Barcelona, Spain) is used to treat difficult warts in humans. In this study, it was applied to nodules that grossly appeared as sebaceous gland hyperplasia. The treatment was carried out on awake dogs. Eleven dogs with a total of 29 sebaceous gland nodules were included in this study. Eighteen of the nodules were treated, and 11 were not treated as a comparison. Four weeks after one treatment, 17/18 of the treated nodules were gone. There was a statistically significant difference in the percentual volume change from day 0 to day 28 between the treated and untreated sebaceous gland nodules. There were no serious side effects. Both the owners of the dogs and the veterinarians were satisfied with this treatment. Verrutop^®^ is an efficient and easy way to remove sebaceous gland hyperplasia in dogs, with minimal side effects.

**Abstract:**

Nodular sebaceous gland hyperplasia in the often middle-aged to old dog is a common, benign proliferation that results in exophytic, pink to yellow, alopecic, and often multilobulated nodules. Removal is usually carried out by surgical excision. As many old dogs have comorbidities that increase the risk of anesthesia, a topical treatment is warranted. We hypothesized that the application of a solution containing nitric acid, zinc, copper, and organic acids (Verrutop^®^), would be a safe and efficient way to treat these nodules. Eleven dogs with a total of 29 nodules, grossly compatible with nodular sebaceous gland hyperplasia, were included in the study. Eighteen of the nodules were treated; 11 were left untreated. No anesthesia or sedation was needed. Four weeks after one application, 17/18 treated nodules had decreased by 100% in volume. There was a statistically significant difference in percentual volume change between the treated and untreated nodules from day 0 to day 28 (*p* < 0.0001). No serious side effects were noted. Sebaceous hyperplasia cannot always be distinguished grossly from sebaceous tumors. Cytological evaluation can be helpful, and in cases of deviant macroscopic features, local recurrence, or more aggressive behavior, the appropriate intervention would be to biopsy or excise the nodule for histopathology. Topical application of Verrutop^®^ is an easy, low-cost, and efficient way to remove canine sebaceous gland hyperplasia with minimal side effects in cases where surgery and anesthesia are not desired.

## 1. Introduction

Sebaceous glands are part of the pilosebaceous unit and, as such, are distributed throughout the haired skin and mucocutaneous junctions of dogs and cats [1]. They are multilobulated, alveolar glands under hormonal influence [2,3,4] that produce lipid-rich sebum, which is essential for epidermal barrier function.

The reported prevalence of tumors of sebaceous gland origin varies between studies and regions [5,6,7,8,9], but they are among the most common skin tumors in older dogs [10,11], while they are uncommon in cats [12].

Proliferations derived from sebaceous glands can be classified as nodular sebaceous hyperplasia, sebaceous adenoma, sebaceous ductal adenoma, sebaceous epithelioma, or adenocarcinoma [4]. Mixed forms also exist [5].

Nodular sebaceous gland hyperplasia is the most common form in dogs [12,13]. It is a benign proliferation that results in exophytic, pink to yellow, sometimes waxy, alopecic, and often multilobulated nodules. They can be up to several centimeters in diameter [12] but are usually under 5 mm [10,14]. They are well circumscribed, often have a central umbilication, and may or may not be ulcerated [5,10,12,14]. Male dogs of toy breeds are reported to be overrepresented [15]. Other common breeds are beagles, cocker spaniels, poodles, dachshunds, and miniature schnauzers [12]. Sebaceous hyperplasia nodules may be single or multiple, and can appear anywhere on the body, but the sites that are commonly affected are the head, chest, and abdomen [15]. In a typical histological examination, the sebaceous lobules are hyperplastic and multiple but otherwise normal in architecture and content, with a layer of basaloid reserve cells in the periphery. The lobules surround one or several widened sebaceous ducts and hair follicles, which may be hyperplastic and dilated. Keratinized squamous epithelium outlines the ducts [14].

Sebaceous adenomas are benign tumors that can be identical with nodular sebaceous hyperplasia, albeit sometimes melanotic [14], with the eyelids and limbs as common locations [12].

Sebaceous epitheliomas are considered low-grade malignant tumors, with mainly the risk of local infiltration and occasionally lymph node metastasis [4], but widespread metastasis has been reported [16]. They may be ulcerated [2], are frequently melanotic [4,12], and most often appear on the eyelids, head, and dorsum [12,14]. There has been some confusion in the distinction between different forms of sebaceous proliferations in the literature [2,5,11,14,17,18]. Sebaceous epithelioma is a variant of basal cell tumor, with a predominance of basaloid cells rather than mature sebaceous cells [13]. In human medicine, the term sebaceoma is used for benign sebaceous neoplasms, where more than 50% of the cells are of basal cell origin, whereas sebaceous adenoma is used if less than 50% are of basal cell origin [19]. The histological features of epitheliomas in veterinary medicine may be assessed as low-grade sebaceous carcinomas by human pathologists [13,17].

Sebaceous carcinomas are uncommon to rare in dogs [10,14,15,20] and often exhibit a different clinical presentation and behavior. They can grow aggressively [15], are usually larger (2.5–7.5 cm in diameter) nodular lesions [12,21], and the epidermis is often ulcerated [2,12]. There may be an increased risk in the cocker spaniel [12], Cavalier King Charles Spaniel, and terrier breeds [18]. They are mainly locally infiltrative, and metastasis rarely occurs [4,12,14,20].

Even if the appearance of sebaceous gland hyperplasia is typical, these nodules cannot always be distinguished from sebaceous tumors. The characteristics of canine benign sebaceous nodules have been evaluated with a dermoscope, which is frequently used to assess sebaceous hyperplasia in human medicine [17,22], but no associations could be found between dermoscopic features and the histological type of sebaceous lesions in dogs [5]. Of thirty-four examined cases of canine sebaceous proliferations, collected and excised over five years, thirty of them were histologically confirmed as sebaceous gland hyperplasia, two were sebaceous adenomas, and two were sebaceous epitheliomas. Three of these thirty-four nodules were mixed forms, classified based on the major type of lesion. Two of the nodules were a mix between sebaceous hyperplasia and adenoma and one was a mix between epithelioma and sebaceous hyperplasia, indicating that sebaceous gland hyperplasia has the potential to undergo neoplastic transformation. There was no case of sebaceous carcinoma [5]. Of 11,740 canine skin tumors registered in the Swiss Canine Cancer Registry between 2008 and 2013, 3.32% were sebaceous gland adenomas, 1.7% were sebaceous gland epitheliomas, and 0.26% were sebaceous gland adenocarcinomas. Cases of sebaceous gland hyperplasia were not included [23]. When ten worldwide studies were collated to determine the overall incidence of the most common canine skin tumors, sebaceous adenoma and hyperplasia were grouped together on place number five with an incidence of 6.5%. The incidence of sebaceous adenocarcinoma was 0.5% [18].

Nodular sebaceous gland hyperplasia, due to its benign nature, does not automatically warrant removal. However, it can cause discomfort if it becomes inflamed, for example, from repeated friction or scratching. With their often multilobulated structure, they may also accumulate debris and microorganisms. Furthermore, lesions can be cosmetically disturbing, which, in the authors’ experience, often motivates owners to request their removal. This is usually carried out by surgical excision [12]. Other less available methods include carbon dioxide laser under local anesthesia [24] or cryotherapy [25].

Older dogs with sebaceous gland nodules often have comorbidities and may be at increased risk for anesthesia, making such procedures undesirable. In addition, although sole lesions occur, it is not uncommon for multiple sebaceous gland hyperplasias to gradually develop over a protracted period of time [12,13]. This could warrant multiple procedures and anesthesia.

In humans, sebaceous gland hyperplasia occurs spontaneously, mostly in the elderly population [26]. Many treatment modalities are used—among them laser, shaving, cryotherapy, excision, and photodynamic therapy [27]. Medical treatment with topical retinoids and oral isotretinoin has also been evaluated [27,28,29]. Many of these treatments are not available in general veterinary practice, require sedation, or, like synthetic retinoids, carry the risk of potentially severe side effects [30]. A topical medical treatment, which has negligible cutaneous and systemic adverse effects, is therefore highly desirable as an alternative.

In human medicine, a nitric zinc complex solution containing nitric acid, zinc, copper, and organic acids (acetic acid, oxalic acid, and lactic acid), with the trade name Verrutop^®^, (manufacturer ISDIN, Barcelona, Spain), is used to treat difficult palmoplantar and small anogenital warts propagated by the human papilloma virus [31,32,33,34,35]. Upon application, the proteins are denaturized by nitric acid, and there is mummification of the tissue. This results in detachment of the wart-like lesion [29] without affecting the skin underneath [34]. It has also been evaluated in the pediatric population with high tolerability [36].

The aim of this prospective, open study was to evaluate the effect of one to two treatments with the mentioned nitric zinc complex solution (Verrutop^®^) on nodular lesions, which were grossly compatible with sebaceous gland hyperplasia in dogs. We hypothesized that this treatment would be successful in macroscopically eliminating the proliferative tissue.

## 2. Materials and Methods

Dogs with one or several nodules clinically compatible with sebaceous gland hyperplasia were included in this study. The inclusion criteria were a gross appearance of exophytic, small (<1 cm in diameter and height), alopecic, not ulcerated, pink to yellow nodules. If a dog had several sebaceous nodules that were deemed eligible for inclusion, around half of them were selected for treatment. The selection was based on the owners’ preferences, which were often those that were most bothersome or cosmetically undesirable. The rest were left untreated as controls. If there was only one sebaceous nodule, that one was treated. Most participants were recruited when visiting or contacting the specialist dermatology department in an animal hospital in Sweden for other reasons. Some were referred by colleagues in other departments who were aware of this study. Cases were included between May 2023 and October 2023, and the assessments were carried out by two board-certified veterinary dermatologists and one European resident in dermatology. If a nodule did not have a clinical appearance compatible with sebaceous gland hyperplasia, the dog was excluded from the study, and the owner was offered a fine needle aspirate for cytological examination or a biopsy for tissue histopathology and further workup. Other exclusion criteria were if the nodule had been treated with any medical product topically within two weeks prior to the first treatment, such as topical corticosteroids. Systemic treatments for unrelated conditions or previous shampooing did not exclude participation in the study.

At the first visit, data regarding breed, age, number of lesions, and how long they had been there, if the dog was disturbed by them, other medications, and any other diseases was collected. A clinical examination was performed, and the nodules were assessed in appearance, photographed, and measured with a caliper.

The hair around the sebaceous nodule was clipped if necessary for photographic reasons, but the skin was not prepared in any way. The aqueous solution (Verrutop^®^) comes in glass ampoules with a capillary tube that serves as an applicator. The tube was dipped into the ampoule to fill it with the solution. The capillary tube was then applied repeatedly with light pressure to the sebaceous nodule, so that a small dot of the solution was expressed. This procedure was repeated at adjacent sites until the entire surface of the nodule was covered with solution and it became gray and shrunken. No sedation or local anesthetic was used. The owner was told to monitor for any swelling, pain, or discharge and returned for a recheck 14 (±3 days) and 28 days (±3 days) after the first treatment. If there was an incomplete regression after 14 days, a second application of Verrutop^®^ was planned to be performed. At the second and third visits, eventual side effects were recorded. The volume of the sebaceous gland nodules was calculated at day 1, before treatment, and at day 28.

The treated and untreated nodules were analyzed as separate samples. The Mann–Whitney test was used to compare the difference in volume change between the treated nodules at day 1 and day 28 with the untreated at the same time interval. The primary outcome was the efficacy of treatment, defined as complete regression macroscopically.

Secondary outcomes were how satisfied the dog’s carer and the veterinarian were with the treatment. The carer was asked to assess the overall satisfaction and success with the treatment on a scale from 0 to 10 at the last visit, where 0 equals no satisfaction and 10 is excellent satisfaction. For the veterinary assessment, a grading scale from 0 to 4 was used, where 0 equals no improvement, 1 equals 25% improvement, and 4 is 100% improvement.

The sample size was determined based on the results of a Swedish pilot study where dogs with nodules macroscopically resembling sebaceous gland hyperplasia or adenomas were treated with a similar, but no longer existing, aqueous formulation containing nitric acid, potassium nitrate, zinc nitrate, and binder (trade name: Oxalic^®^) [37]. Power was set to 90%, and a *p*-value < 0.05 was considered significant. All owners signed informed consent about the study and could withdraw at any time. This study was ethically approved in accordance with Swedish law number SJVFS 2019:9, L150 §18.

## 3. Results

Twelve dogs met the inclusion criteria. They had one to six sebaceous gland nodules each. The breeds that were included were mixed breeds (*n* = 4), bichon frisé (*n* = 1), bichon havanais (*n* = 1), Cavalier King Charles Spaniel (*n* = 1), miniature poodle (*n* = 1), Yorkshire terrier (*n* = 1), Australian shepherd (*n* = 1), Border collie (*n* = 1) and miniature bullterrier (*n* = 1). Their age was 4–14 years, with a median age of 9.5 years and a mean age of 9.3 years. There were five neutered males, three males, three bitches, and one spayed female. Six dogs had atopic dermatitis. One was hypothyroid and had chronic gastrointestinal disease, tracheal collapse, and degenerative mitral valve disease. There was one dog with idiopathic epilepsy, one with pyoderma, and one with a mammary tumor. One dog had just been treated for neck pain; another had osteoarthritis. Two dogs had no comorbidity (Table 1). The sebaceous gland nodules were discovered by the owner from two years to a few days before inclusion. Most dogs were not disturbed by their lesions, but one repeatedly got stuck in the harness, and another used to bleed when the dog scratched it. One case (a male Yorkshire terrier) was not able to travel to the clinic again after the first visit and was excluded from the analysis.

A total of 18 sebaceous gland nodules were treated with Verrutop^®^, and 11 were left untreated. The treated nodules varied in volume between 18 mm^3^ and 168 mm^3^ with a mean of 61.8 mm^3^ (SD 43.7) and a median of 41 mm^3^ before treatment. The untreated ones varied in volume between 6 mm^3^ and 112 mm^3^, with a mean of 42.5 mm^3^ (SD 38.0) and a median of 18 mm^3^ on the day of inclusion. There was no statistically significant difference in the mean size between the treated and untreated nodules at inclusion day before treatment (*p* = 0.23).

**Figure 1:**
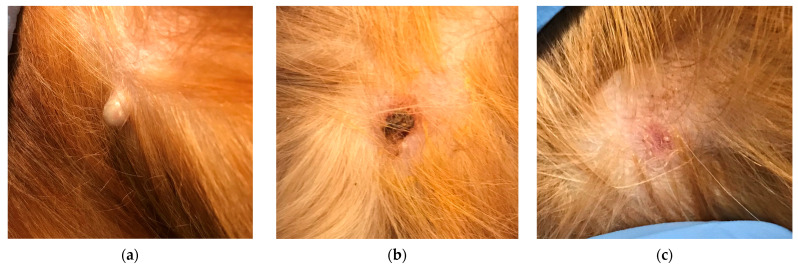
Sebaceous gland hyperplasia at inclusion day 1 (**a**), day 14 (**b**), and day 28 (**c**) after one treatment with Verrutop^®^. The volume decreased by 100%, with only mild scaling and erythema visible at day 28.

**Figure 2:**
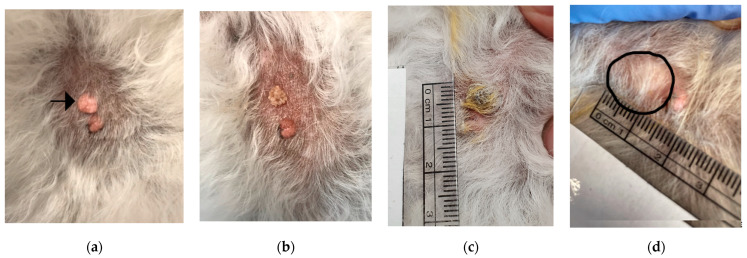
Two sebaceous nodules on a dog. The upper nodule in the picture (arrow) was treated with Verrutop^®^; the one caudal of this was left untreated as a control. Pictures from left to right: before treatment (**a**); directly after the application of Verrutop^®^ on the upper one (**b**); after two weeks, there is a scab in the fur and yellow coloring of the fur (**c**); after 4 weeks; the treated skin is flat and unremarkable (**d**, encircled area), and the untreated nodule remains unchanged.

**Figure 3:**
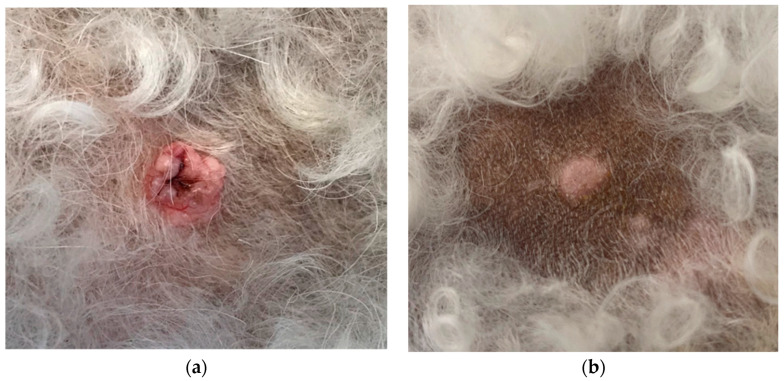
One sebaceous gland hyperplasia at day 1 before treatment (**a**) and day 28, after the treatment with Verrutop^®^ (**b**). The surface is smooth, but a slight elevation of the skin remains. The volume of the original nodule decreased by 83%.

**Figure 4:**
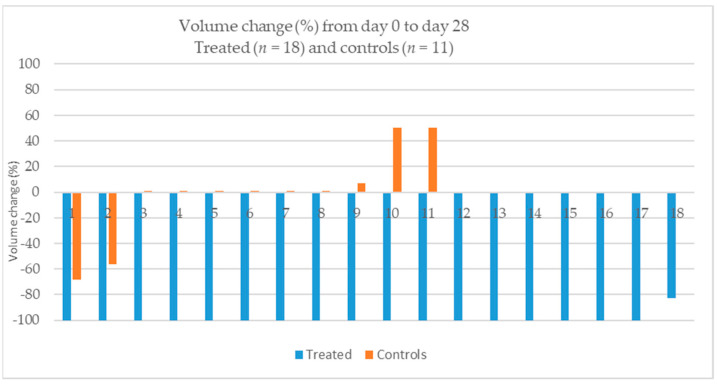
Percentual volume change for treated and untreated (controls) nodules from day 0 to day 28.

For 17 of the 18 treated nodules, one single Verrutop^®^ treatment led to complete regression (Table 2; Figure 4; see examples in Figure 1 and Figure 2), and the remaining one decreased in volume by 83% (Figure 3).

Of the 17 nodules that were completely resolved, one had a thin crust over the treated area at day 28, and one had erosion. None of the treated nodules were assessed as requiring an additional/second treatment.

At day 28, the volume of six of the eleven nodules that did not receive any treatment was identical to that at day 0. One had decreased by 68% in volume, and another by 56%. One had increased by 7%, and two had increased by 50% (Table 2; Figure 4). There was a statistically significant difference in percentual volume change from day 0 and day 28 between the treated and untreated sebaceous gland nodules (*p* < 0.0001).

Of eleven treated dogs, side effects were reported in four. One dog reacted during the application of the solution as if it were uncomfortable or painful. A transient licking of the area was observed in two dogs. One dog vomited once the night after the treatment.

Nine of eleven owners regarded the success and satisfaction with the treatment as 10/10 when they were asked to grade it from 0 to 10 at day 28. One owner graded the treatment as 9/10 and one as 8/10. The veterinarians assessed the effect as 100% (4 on a scale of 0–4) in nine out of eleven treated dogs and as 75% (3 on a scale of 0–4) in two cases.

## 4. Discussion

This study demonstrated that one topical treatment with Verrutop^®^ was able to macroscopically eliminate sebaceous gland nodules in dogs effectively and with minimal side effects. The treatment protocol is highly cost-effective, can be performed by general practitioners, and does not require any advanced equipment. The solution was easily applied to the proliferative lesions without any need for local or general anesthesia or sedation. In the treatment group, one single Verrutop^®^ treatment achieved complete resolution for 17 of 18 nodules. In the one that did not respond completely, a slight elevation of the skin surface remained. Yet, it was decided not to perform a second treatment, as the owner was happy with the effect.

The side effects were minor. In general, the local fur and skin get transiently colored yellow after treatment. The majority of dogs did not react at all to the Verrutop^®^ application, leading us to conclude that it was painless. However, one dog reacted as if it was uncomfortable. To mitigate any potential painful stimulus, lidocaine cream could be used topically before application.

Two dogs licked the treated area afterwards. One kept doing it frequently for two weeks, and then it ceased without intervention. One dog licked the treated area on and off for five weeks after the application, indicating pruritus or pain. There was no swelling or discharge. At the third visit, four weeks after the treatment, the skin was unremarkable but still mildly pruritic. Hydrocortisone cream (10 mg/g) was applied to the area for seven days, and the behavior stopped eventually. The carer rated the satisfaction and success of treatment as 8/10 (the pain/pruritus lowered the score). One dog vomited once the night after the application of Verrutop^®^. Whether or not this was associated with the treatment or if it was an unrelated event was not elucidated. The owner had not seen the dog lick the area, but it cannot be discounted that the licking had occurred unnoticed and had triggered the vomiting. The owner reported the incident to the dermatology department, but no veterinary intervention was carried out as it was a single episode and the dog appeared normal afterwards.

Most of the dogs in this study were middle-aged or older and had comorbidities. Even if these dogs’ underlying conditions did not make them directly unsuitable for surgery, anesthetizing an older patient may also require blood work or other perioperative measures, which increase the time and cost of the intervention. These factors can also motivate owners to seek an alternative to surgery.

Due to their comorbidities, some of the dogs in the study were on systemic medications (Table 1). Even if concurrent medication in theory could be a confounder, it was deemed unlikely that it would affect the outcome of the topical Verrutop^®^ treatment. In addition, the inclusion of untreated, grossly identical lesions on the same dog as controls should have decreased that risk.

The treatment results of this study are in accordance with a Swedish pilot study where nodules macroscopically compatible with sebaceous gland hyperplasia were treated once or twice with a solution containing nitric acid, potassium nitrate, zinc nitrate, and binder (Oxalic^®^). After 28 days, 91.3% of the treated lesions were gone [37]. In another, similar pilot study, 83% of veterinarians and 89% of owners regarded the response to the same treatment as excellent [38]. Comparably, a study with cryotherapy to remove benign skin tumors (mostly sebaceous adenomas) on awake animals had a success rate of 57%, and 33% of the dogs showed pain during the treatment session [25]. In human medicine, nitric zinc complex solution (Verrutop^®^) has been compared to cryotherapy for treating anogenital warts, with a good effect, better tolerability, and a lower recurrence rate [34]. In plantar warts, other studies show similar results as treatment with cryotherapy [35].

Most of the dogs in this study were not disturbed by their sebaceous gland nodules before treatment. This is in contrast to the Swedish pilot study, where eight of thirteen dogs were reported to have been licking or scratching their sebaceous proliferations [37]. This difference could mirror the recruitment process. In the pilot study, owners actively responded to a call for participants among breeding clubs, which likely resulted in an overrepresentation of dogs whose owners considered the nodules to be bothersome. In this study, the majority of the dogs were included when they visited the dermatology department for reasons other than the lesions themselves, such as managing atopic dermatitis. For most of these owners, the alternative to treating the nodules topically was to only observe them.

The main inclusion criteria that patients’ proliferative lesions needed to fulfill was that they were clinically compatible with sebaceous gland hyperplasia (described under the Materials and Methods Section). For this study, we elected to use solely macroscopic appearance and not microscopic assessment. Judging on the prevalence of the varying types of sebaceous gland proliferations, we expect that the majority of the nodules were sebaceous gland hyperplasia, but we cannot exclude that some of them were sebaceous adenomas or possibly epitheliomas. Cytological evaluation can be diagnostic, but histopathology may be required to determine the type of sebaceous proliferation [39]. We wished to assess the validity of a treatment that requires minimal intervention and its suitability in general practice, where funds as well as specialized equipment may be lacking. Hence, neither fine needle aspiration nor biopsies of lesion tissue were performed in this study. As we cannot be sure that we have exclusively treated sebaceous gland hyperplasia, this is a major limitation of the study. The results show clearly, though, that lesions grossly compatible with sebaceous gland hyperplasia respond well to Verrutop^®^. Clinically, it is not crucial to distinguish sebaceous gland hyperplasia from sebaceous adenoma, as both lesions are benign [18]. But if an epithelioma is treated with Verrutop^®^, there may be a risk of local recurrence and potentially malignant behavior [11]. The overall risk of serious consequences if mistaking a sebaceous epithelioma for sebaceous gland hyperplasia should be low, as severe malignant behavior of the former is rare [13,14,15]. But to minimize that risk, a fine needle aspirate could be taken before the decision to apply Verrutop^®^. It should also be emphasized that in any case of uncertainty, different macroscopic features, local recurrence, or more aggressive behavior, the appropriate intervention would be to biopsy or excise the nodule for histopathology.

Another limitation of this study is that the recurrence rate is unknown, as no long-term follow-up has been conducted. The effect in the short term is very convincing, but future studies should include more dogs, cytological evaluation, and longer follow-up. This study also provides the basis for further research to assess whether Verrutop^®^ is suitable as a medical treatment for other types of cutaneous benign tumors and hyperplastic lesions.

The solution with a nitric zinc complex is of great value when dealing with difficult palmoplantar and anogenital warts in human medicine [31,32,33,34,35,36]. This study shows that it can also be of use in veterinary medicine. The application of Verrutop^®^ is an easy and effective way to remove nodules clinically compatible with canine sebaceous gland hyperplasia in cases where surgery and anesthesia are not a desired option.

## 5. Conclusions

A single topical application of a solution with nitric acid, zinc, copper, and organic acids (Verrutop^®^) is an efficient and easily performed treatment with minimal side effects to remove nodules grossly compatible with sebaceous gland hyperplasia in dogs. The treatment is well tolerated and can be performed by general practitioners in cases where removal is required, but medical or other reasons make surgery and anesthesia undesirable.

## Figures and Tables

**Table 1:** Data of the dogs enrolled in the study, with the comorbidities, treatments, and number of treated or untreated sebaceous nodular proliferations.

Breed	Sex	Age	Weight	Concurrent Disease	Systemic Treatment	Treated (*n* = 18)	In Remission at Day 28 Total: 17/18	Untreated (*n* = 11)	In Remission at Day 28 Total: 0/11
Bichon frisé	NM	7 y	9.3 kg	None	None	2	1/2	2	0/2
Miniature bullterrier	NM	10 y	20.2 kg	cAD; pododermatitis; overweight	Methylprednisolone (0.2 mg/kg) every other day	1	1/1	0	NA
Bichon havanais	F	8 y	6.9 kg	cAD; mammary tumor	Lokivetmab (1.4 mg/kg) in irregular intervals; subcutaneous allergen specific immunotherapy	2	2/2	2	0/2
Border collie	NM	4 y	19.5 kg	None	None	2	2/2	2	0/2
Australian shepherd	NM	8 y	26.2 kg	Idiopathic epilepsy	Potassium bromide (31 mg/kg) q 24 h; phenobarbital (3.8 mg/kg) q 12 h	1	1/1	0	NA
Cavalier King Charles Spaniel	F	9 y	14.5 kg	cAD, food allergy; osteoarthritis	Lokivetmab (2.1 mg/kg) every 4–5 months; bedinvetmab (0.69 mg/kg) monthly; firocoxib (5.9 mg/kg) daily	1	1/1	0	NA
Mixed breed	NM	9 y	9.4 kg	cAD	Subcutaneous allergen specific immunotherapy	2	2/2	1	0/1
Mixed breed	F	10 y	7.9 kg	cAD; food allergy	Oclacitinib (0.45 mg/kg) q 24 h	1	1/1	0	NA
Miniature poodle	M	14 y	6 kg	Hypothyroid, chronic GI disease; tracheal collapse; MMVD	Levothyroxine (dose unknown)	1	1/1	1	0/1
Mixed breed	SF	11 y	19 kg	Transient lameness and neck pain	Robenacoxib (1 mg/kg), stopped 3 days before inclusion	1	1/1	1	0/1
Mixed breed	M	12 y	24.3 kg	Pyoderma	Amoxicillin (8 mg/kg) q 12 h; cetirizine (2 mg/kg) q 24 h	4	4/4	2	0/2

NM = neutered male; F = female; M = male; and SF = spayed female. y = years. cAD = canine atopic dermatitis. NA = not applicable.

**Table 2:** Percentual volume change for treated and untreated nodules from day 0 to day 28.

Volume Change	−100	−83	−68	−56	0	+7	+50	Total
Treated	17	1						18
Untreated			1	1	6	1	2	11

## Data Availability

The data presented in this study are available on reasonable request from the corresponding author.
